# Passive Homodyne Phase Demodulation Technique Based on LF-TIT-DCM Algorithm for Interferometric Sensors

**DOI:** 10.3390/s21248257

**Published:** 2021-12-10

**Authors:** Wanjin Zhang, Ping Lu, Zhiyuan Qu, Jiangshan Zhang, Qiang Wu, Deming Liu

**Affiliations:** 1 Wuhan National Laboratory for Optoelectronics (WNLO) and National Engineering Laboratory for Next Generation Internet Access System, School of Optical and Electronic Information, Huazhong University of Science and Technology, Wuhan 430074, China; zhangwanjin@hust.edu.cn (W.Z.); d201880622@hust.edu.cn (Z.Q.); dmliu@hust.edu.cn (D.L.); 2 Huazhong University of Science and Technology Research Institute, Huazhong University of Science and Technology, Shenzhen 518000, China; 3 Department of Electronics and Information Engineering, Huazhong University of Science and Technology, Wuhan 430074, China; zhangjs@hust.edu.cn; 4 Faculty of Engineering and Environment, Northumbria University, Newcastle Upon Tyne NE1 8ST, UK; qiang.wu@northumbria.ac.uk

**Keywords:** passive homodyne phase demodulation, linear fitting, trigonometric identity transformation, differential cross multiply, interferometric sensors

## Abstract

A passive homodyne phase demodulation technique based on a linear-fitting trigonometric-identity-transformation differential cross-multiplication (LF-TIT-DCM) algorithm is proposed. This technique relies on two interferometric signals whose interferometric phase difference is odd times of π. It is able to demodulate phase signals with a large dynamic range and wide frequency band. An anti-phase dual wavelength demodulation system is built to prove the LF-TIT-DCM algorithm. Comparing the traditional quadrature dual wavelength demodulation system with an ellipse fitting DCM (EF-DCM) algorithm, the phase difference of two interferometric signals of the anti-phase dual wavelength demodulation system is set to be π instead of π/2. This technique overcomes the drawback of EF-DCM—that it is not able to demodulate small signals since the ellipse degenerates into a straight line and the ellipse fitting algorithm is invalidated. Experimental results show that the dynamic range of the proposed anti-phase dual wavelength demodulation system is much larger than that of the traditional quadrature dual wavelength demodulation system. Moreover, the proposed anti-phase dual wavelength demodulation system is hardly influenced by optical power, and the laser wavelength should be strictly limited to lower the reference error.

## 1. Introduction

Fiber optic sensors have been widely researched because of their advantages such as high sensitivity, light weight, electromagnetic immunity, and multiplexing capabilities [1,2,3,4,5]. Among fiber optic sensors, interferometric sensors play an important role including four types such as Michelson interferometric (MI) sensors [6], Mach-Zehnder interferometric (MZI) sensors [7], Sagnac interferometric (SI) sensors [8] and Fabry–Perot interferometric (FPI) sensors [9]. For interferometric sensors, the signal to be measured is demodulated in a phase of an interferometer, where a specific demodulation method is applied to the interrogation phase signal introduced by the signal to be measured. Several demodulation methods have been reported including homodyne phase demodulation [10,11,12,13,14,15,16,17,18,19,20,21,22], heterodyne phase demodulation [23,24,25] and spectrum demodulation [26,27,28]. Homodyne phase demodulation has attracted much attention due to its large dynamic range, wide frequency band and immunity to phase noise from the laser source. It can be divided into two categories, which are active and passive homodyne phase demodulation. A typical structure of active homodyne phase demodulation is active wavelength tuning homodyne demodulation, which is simple and easy to be realized [10,11,12], whereas the dynamic range of active wavelength tuning homodyne demodulation is limited since a linear approximation of the interferometric function is introduced, which makes it only suitable for small signal demodulation. For passive homodyne phase demodulation, there are several different types, such as 3 × 3 coupler demodulation [13,14], phase generated carrier (PGC) demodulation [15,16,17,18] and quadrature dual wavelength demodulation [19,20,21,22]. The characteristics of 3 × 3 couplers, i.e., that phase difference is 2π/3 and the coupling ration is 1:1:1 to realize phase demodulation, are taken advantage of by 3 × 3 coupler demodulation, which becomes invalid when the characteristics of the 3 × 3 coupler are not ideal. Moreover, 3 × 3 coupler demodulation is only applicable for MI sensors and MZI sensors. PGC demodulation is widely used in MI and MZI sensors because their optical path difference (OPD) is long enough to provide enough modulation depth. On the contrary, PGC demodulation is hardly able to be used in FPI sensors, whose OPD is usually several hundred micrometers. Moreover, the demodulating frequency should be much higher than the frequency of the signal to be measured and the system sampling rate should be much higher than the demodulating frequency, which requires a rather high sampling rate and makes PGC demodulation unsuitable for high-frequency signal demodulation. Quadrature dual wavelength demodulation obtains two orthogonal signals through two different laser wavelengths and uses an ellipse fitting (EF) algorithm to calculate the direct current component for normalization and uses a differential cross-multiplication (DCM) algorithm to interrogate the phase signal from two normalized signals. Compared with PGC demodulation, quadrature dual wavelength demodulation does not need a carrier signal, which expands its frequency band and makes it usable for FPI sensors. However, an EF algorithm is valid and effective when a phase signal is introduced when the signal to be measured is large enough to form an obvious curve from an ellipse. In this case, quadrature dual wavelength is only suitable for large signal demodulation.

In this paper, we put forward a passive homodyne phase demodulation technique based on an LF-TIT-DCM algorithm for interferometric sensors. The LF-TIT-DCM algorithm is applied to two interferometric signals whose interferometric phase difference is odd times of π. It is able to demodulate the phase signal of a large dynamic range and wide frequency band. An anti-phase dual wavelength phase demodulation system is built to prove the LF-TIT-DCM algorithm. Moreover, a traditional quadrature dual wavelength demodulation system based on an EF-DCM algorithm is also built to compare their dynamic range, especially for small signal demodulation. The influence of optical power and wavelength is also researched at different sound pressure levels.

## 2. Materials and Methods

### 2.1. Algorithm Principle

Two interferometric signals whose interferometric phase difference is odd times of π can be expressed as:

(1) $$\begin{array}{l} V_{1}=k_{1}I_{1}[1+B_{1}\cos(\varphi +\varphi (t))]\\ V_{2}=k_{2}I_{2}[1+B_{2}\cos(\varphi +\varphi (t))+(2m+1)\pi ] \end{array}$$

where *k*_1_ and *k*_2_ are related with photoelectric conversion coefficients, *I*_1_ and *I*_2_ are incident intensities to two interferometers, *B*_1_ and *B*_2_ are interferometric contrasts of two interferometers, *φ* is the initial phase and *φ*(*t*) is the phase signal introduced by the external signal to be measured. When plotting two interferometric signals in a coordinate system, a straight line can be obtained. The equation of the straight line can be expressed as:

(2) $$V_{1}+kV_{2}=b.$$

With a linear fitting algorithm such as the least square method, the coefficient of the straight line can be calculated as:

(3) $$\begin{array}{l} k=-\frac{\bar{V_{1}V_{2}}-\bar{V_{1}}\bar{V_{2}}}{\bar{V_{2}^{2}}-{\bar{V_{2}}}^{2}}\\ b=\bar{V_{1}}+k\bar{V_{2}} \end{array}$$

By combining Equations (1) and (2), a normalized signal can be calculated as:

(4) $$V_{n}=\cos(\varphi +\varphi (t))=\left(B_{1}V_{1}-kB_{2}V_{2}\right)/\left(B_{1}B_{2}b\right)$$

Furthermore, by applying trigonometric identity transformation to Equation (4), two orthogonal signals with an absolute value sign can be calculated as:

(5) $$\begin{array}{l} V_{x}=\left|V_{xs}\right|=\left|\cos(\varphi +\varphi (t))/2\right|=\sqrt{\left(1+V_{n}\right)/2}\\ V_{y}=\left|V_{ys}\right|=\left|\sin(\varphi +\varphi (t))/2\right|=\sqrt{\left(1-V_{n}\right)/2} \end{array}$$

Since the absolute value function influences the continuity of the first derivative of the applied function, the absolute value sign can be removed according to this characteristic. After removing the absolute value sign, two orthogonal signals can be obtained. Therefore, the differential cross multiplication method can be applied to these two orthogonal signals to demodulate the phase signal [17]:

(6) $$\varphi (t)=2\int \left(V_{xs}\prime V_{ys}-V_{xs}V_{ys}\prime \right)dt$$

From Equations (4) and (6), it can be concluded that the demodulated phase signal is regardless of optical power or sensitivity of photodetectors. Moreover, owing to usage of the DCM algorithm, the amplitude of the demodulated phase signal is not restricted anymore. Similar to the traditional EF-DCM-based passive homodyne algorithm, the proposed LF-TIT-DCM-based passive homodyne algorithm can demodulate the signal of wide-band frequency as long as the sampling rate of the demodulating system is enough, since there is no frequency limitation during the proposed phase demodulation process. Compared with a traditional EF-DCM-based passive homodyne algorithm, the proposed LF-TIT-DCM-based passive homodyne algorithm can demodulate a small signal since part of a straight line is still a straight line, which means that the linear fitting is still valid. On the contrary, part of an ellipse can be regarded as a straight line which introduces great error for an ellipse fitting algorithm. Moreover, although interferometric contrast of interferometers is introduced, in most cases, the interferometric contrast of an interferometer can be regarded as a constant. As long as an interferometric sensor is fabricated, the reflectivity of the two facets of the interferometric sensor is constant. Furthermore, although interferometric contrast is still impacted by the linewidth of the laser, the influence is limited for most single wavelength lasers whose linewidth is below several megahertz.

Furthermore, there are several ways to obtain two interferometric signals whose interferometric phase difference is odd times of π. Since the interferometric phase of an interferometer is related to its laser wavelength, refractive index and optical path, three possible passive homodyne phase demodulation systems can be put forward according to how the interferometric phase difference is introduced. Among them, the dual wavelength demodulation system is much easier to be realized and can be applied to different types of interferometric sensors.

### 2.2. Dual Wavelength Demodulation System

**Figure 1:**
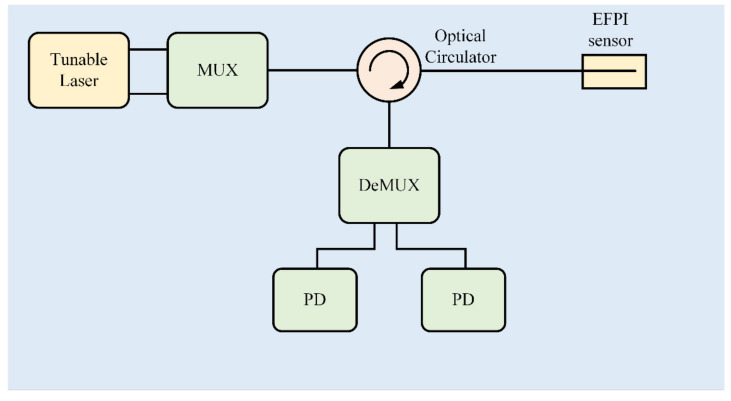
System schematic of dual wavelength demodulation system. MUX: multiplexer, DeMUX: demultiplexer, PD: photodetector.

A typical system schematic of a dual wavelength demodulation is as shown in Figure 1. Two light beams with different wavelengths are emitted from a multi-output tunable laser and are combined by a wavelength division multiplexer (MUX). Then the combined light is injected into an extrinsic Fabry-Perot interferometric (EFPI) sensor through an optical circulator. Reflected light from the EFPI sensor is divided by a wavelength division demultiplexer (DeMUX). The separated light is converted to voltage signals through a photodetector (PD).

Output voltage signals of two photodetectors can be expressed as [22]:

(7) $$\begin{array}{l} V_{1}=k_{1}I_{1}\left[1+B\cos(4\pi nL/\lambda_{1}+\varphi (t))\right]\\ V_{2}=k_{2}I_{2}\left[1+B\cos(4\pi nL/\lambda_{2}+\varphi (t))\right] \end{array}$$

where *B* is the interferometric contrast of the EFPI sensor, *n* is the refractive index, *L* is the cavity length and *λ*_i_ (i = 1, 2) is the laser wavelength. The interferometric phase difference of two voltage signals can be expressed as:

(8) $$\Delta \varphi =4\pi nL\left(\frac{1}{\lambda_{1}}-\frac{1}{\lambda_{2}}\right)\approx \frac{2\pi \Delta \lambda }{FSR}$$

where *FSR* is the free spectrum range of the EFPI sensor and can be obtained through an optical spectrum analyzer. Through controlling the laser wavelength difference of two lasers from the tunable laser, the phase difference can be tuned to be odd times of π, where the relationship between laser wavelength difference and *FSR* can be expressed as:

(9) $$\Delta \lambda =\frac{2m+1}{2}FSR$$

where *m* is an integer.

In this case, through controlling the laser wavelength difference of two lasers from the tunable laser to fulfill Equation (9), two interferometric signals can be obtained and the LF-TIT-DCM algorithm can be applied to the proposed dual wavelength demodulation system. Moreover, by controlling the laser wavelength difference to make the interferometric phase difference to be odd times of π/2, the traditional EF-DCM-based quadrature dual wavelength demodulation system is achieved. Since the cavity length of EFPI acoustic sensor is easily influenced by temperature, the *FSR* of the EFPI acoustic sensor is influenced by temperature. In this case, the interferometric phase difference of two voltage signals changes with temperature. However, this influence is limited. According to Equation (8) and the computational formula of *FSR* = *λ*^2^/2*nL*, the relative variation of phase difference equals the relative variation of the cavity length, which is *d*Δ*φ*/Δ*φ* = *dL*/*L*. For EFPI acoustic sensors whose cavity lengths are several hundred microns, the variation of cavity length introduced by temperature is below several microns. In this case, the relative variation of phase difference introduced by the temperature is below 1%, which is negligible. It can be concluded that influence of temperature is limited.

## 3. Results and Discussion

### 3.1. Acoustic Signal Testing System

**Figure 2:**
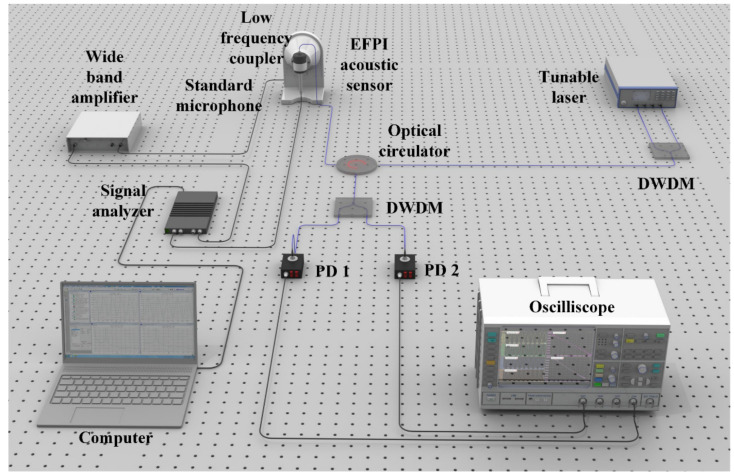
Acoustic signal testing system.

To prove the aforementioned theory, an EFPI acoustic sensor is fabricated and an acoustic signal testing system is built. The EFPI acoustic sensor is comprised of a vibrating diaphragm with a fixed boundary and an optical fiber which is perpendicular to the diaphragm to form an extrinsic Fabry–Perot interferometer. The *FSR* and the interferometric contrast of the EFPI acoustic sensor are 2.188 nm and 0.992, respectively. The acoustic signal testing system is shown in Figure 2. Two light beams are emitted from a multi-output tunable laser (Alnair Labs, TLG 210, Tokyo, Japan), whose wavelengths are 1555.682 nm and 1556.776 nm, respectively. Two light beams are combined together through a dense wavelength division multiplexer (DWDM). The combined light enters into the EFPI acoustic sensor through an optical circulator. Reflected light from the EFPI acoustic sensor is separated by another dense wavelength division multiplexer with the same parameter as the first one. The separated light is converted to voltage signals by two photodetectors (New Focus, 1623, California, CA, USA), PD1 and PD2, respectively. Output voltage signals from the two photodetectors are collected by an oscilloscope (Rohde and Schwarz, RTE 1054, Munich, Germany) and converted to digital signals. The EFPI acoustic sensor is sealed in a low-frequency coupler (Brüel and Kjær, WB-3570, Nærum, Denmark) where the acoustic signal is generated, and a standard microphone (Brüel and Kjær, 4193-L-004, Nærum, Denmark) is used to calibrate amplitude of the acoustic signal. A signal analyzer (Brüel and Kjær, 3160 PULSE LAN-XI, Nærum, Denmark) is used to output the driving signal, which is amplified by a wide-band amplifier (Brüel and Kjær, WQ-3205, Nærum, Denmark). The calibrated signal from the standard microphone is also collected by the signal analyzer, which is controlled through a computer.

### 3.2. Small Signal and Large Signal Response

**Figure 3:**
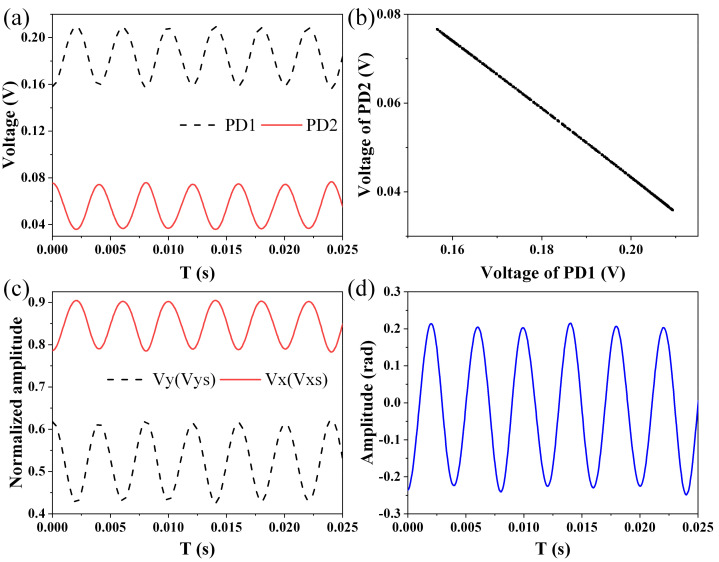
Experimental results in small signal condition. (**a**) Output signals from two photodetectors. (**b**) Scatter diagram of output signals. (**c**) Orthogonal signals. (**d**) Demodulation phase signal.

**Figure 4:**
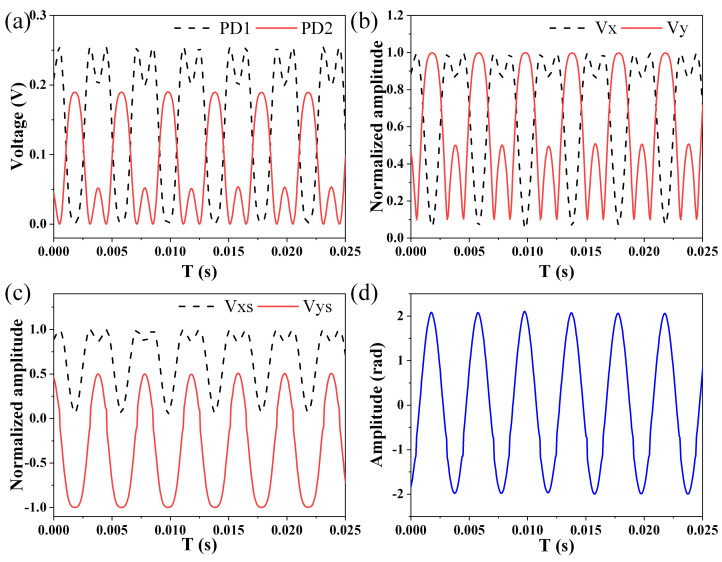
Experimental results in large signal condition. (**a**) Output signals from two photodetectors. (**b**) Orthogonal signals before removing absolute value sign. (**c**) Orthogonal signals after removing absolute value sign. (**d**) Demodulation phase signal.

First of all, a small signal and a large signal are applied to the EFPI acoustic sensor respectively to prove that the proposed LF-TIT-DCM-based passive homodyne phase demodulation is suitable for both small signal and large signal demodulation. Experimental results are shown in Figure 3 and Figure 4.

Figure 3a shows the output signals from the two photodetectors in the small signal condition. Since the amplitude of the applied acoustic signal is small, the output signals are proportionate to the acoustic signal, and the phase difference of them is π. Figure 3b shows a scatter diagram of these two output signals. All of the points are located on a straight line. Even if the amplitude of the applied acoustic signal is smaller, as long as the precision of the oscilloscope is high enough and the system noise is low enough, the straight line always exists and becomes shorter instead of shrinking into a point. It affirms that the linear fitting algorithm can work properly no matter how small the amplitude of the applied acoustic signal is, which makes the proposed LF-TIT-DCM-based passive homodyne phase demodulation perform better than the EF-DCM-based passive homodyne phase demodulation. Figure 3c shows two orthogonal signals calculated from a normalized signal. Since the orthogonal signals are small enough, there is no difference before and after removing the absolute value sign. Figure 3d shows the demodulated phase signal calculated from those two orthogonal signals using the DCM algorithm and the phase amplitude is calculated to be 0.22 rad.

Figure 4a shows output signals from the two photodetectors in the large signal condition. Since the phase amplitude introduced by the applied acoustic signal is large enough, significant distortion is observed in these two signals because the phase amplitude exceeds the linear region of interferometric function, which is known as cosine function. The distortions in the two signals are opposite to each other since their phase difference is equal to π. Moreover, these distortions mean that the output signals have a much more harmonic component, which requires a higher sampling rate for the oscilloscope. The larger the phase amplitude is, the higher the sampling rate should be. Two orthogonal signals before and after removing the absolute value sign are shown in Figure 4b,c, respectively. In the large signal condition, the process of removing the absolute value sign is vital to obtain two ideal orthogonal signals. Figure 4d shows the demodulated phase signal calculated from those two orthogonal signals with the DCM algorithm and the phase amplitude is calculated to be 2.03 rad.

### 3.3. Dynamic Range and Comparison

**Figure 5:**
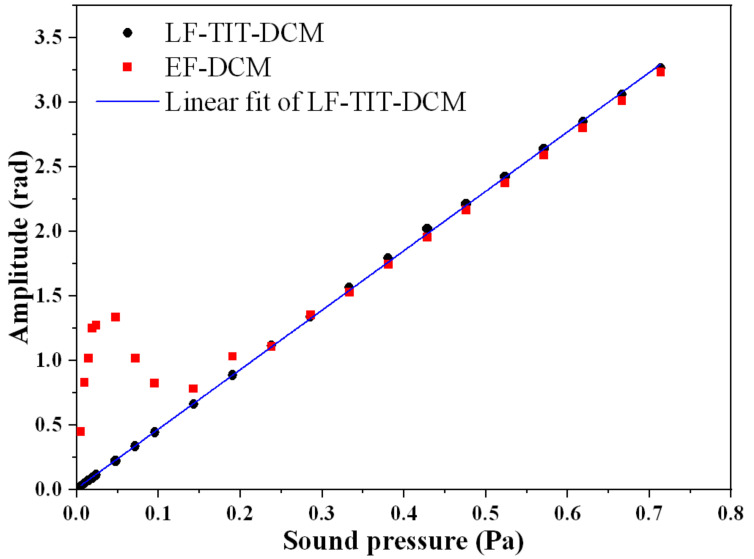
Comparison of linear response between LF-TIT-DCM-based passive homodyne phase demodulation and EF-DCM-based passive homodyne phase demodulation.

To prove that the proposed LF-TIT-DCM-based passive homodyne phase demodulation performs better than the EF-DCM-based passive homodyne phase demodulation, a comparison of the linear response region is made. For the EF-DCM-based passive homodyne phase demodulation, the phase difference of the two output signals is π/2 instead of π. In this case, the laser wavelengths of the two output ports of the tunable laser are set as 1554.784 nm and 1556.425 nm, respectively. The amplitude of the applied acoustic signal varies from 0.005 Pa to 0.714 Pa. The experimental result is shown in Figure 5. When the amplitude of the applied acoustic signal is larger than 0.24 Pa, the demodulated phase amplitudes of two algorithms are nearly the same as each other and show excellent linearity, which is consistent with the aforementioned theory. On the contrary, when the amplitude of the applied acoustic signal is smaller than 0.24 Pa, a great error is observed for the EF-DCM-based passive homodyne phase demodulation, which is mainly caused by an error from the ellipse fitting algorithm. This proves that the ellipse fitting algorithm becomes invalid when the phase amplitude is too small to form an ellipse instead of a straight line. The phase sensitivity of the EFPI acoustic sensor calculated from the LF-TIT-DCM-based passive homodyne phase demodulation is 4.61 rad/Pa. Moreover, linearity is as high as 0.9998. For the EF-DCM algorithm, a major error is introduced from the EF algorithm, especially in the small signal condition, since an ellipse degenerates into a straight line when the phase amplitude is small enough. For the LF-TIT-DCM algorithm, a major error is introduced from the phase difference of those two interferometric signals. When the phase difference deviates from π, the straight line will transform into an oblate ellipse, which introduces an error for the LF algorithm. Both of these two algorithms are rid of the influence of the fluctuation of laser power.

### 3.4. Optical Power Response

**Figure 6:**
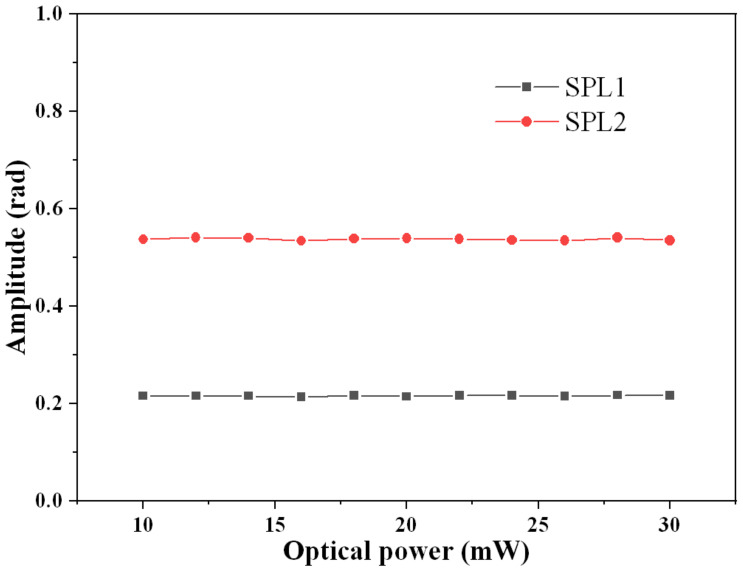
Relationship between optical power and demodulated phase amplitude. SPL1: 67.6 dB, SPL2: 75.6 dB.

Moreover, the influence of optical power and laser wavelength are researched. The relationship between optical power and the demodulated phase amplitude is shown in Figure 6. Experiments are performed at two different sound pressure levels (SPL), which are 67.6 dB (SPL1) and 75.6 dB (SPL2) re 20 μPa, respectively. Optical power varies from 10 mW to 30 mW. Maximal relative errors at SPL1 and SPL2 are calculated to be 0.8% and 0.6%, respectively. Although the two output signals from the two photodetectors are influenced by optical power, the normalized signal calculated from those two output signals is hardly influenced by optical power, which ensures that the demodulated phase signal is irrelevant with optical power. Furthermore, no matter how large the SPL is, the demodulated phase amplitude is hardly influenced by optical power. Both of these two experimental results are consistent with the aforementioned theory. In this case, the proposed LF-TIT-DCM-based passive homodyne phase demodulation can overcome the drawback that intensity-based quadrature point demodulation is influenced by optical power. Meanwhile, the optical power should not be too small since smaller output signals from photodetectors require higher sampling precision of the oscilloscope.

### 3.5. Wavelength Response

**Figure 7:**
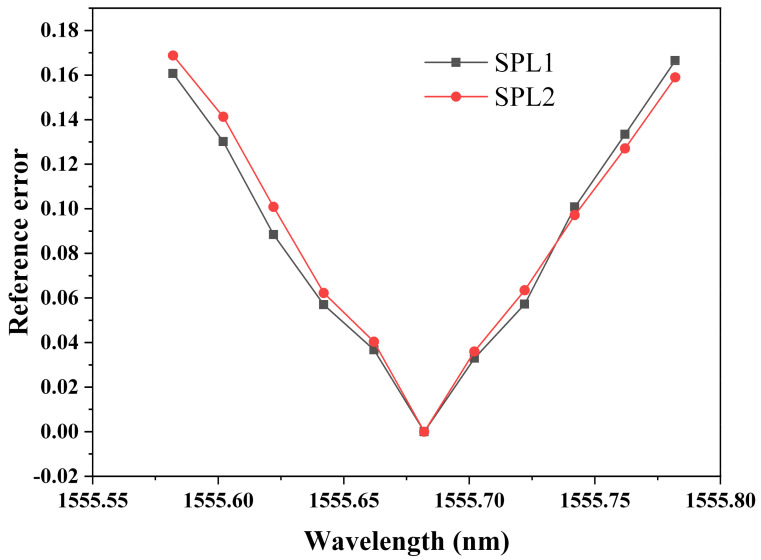
Relationship between laser wavelength and reference error of demodulated phase amplitude. SPL1: 67.6 dB, SPL2: 75.6 dB.

The relationship between laser wavelength and reference error of the demodulated phase amplitude is shown in Figure 7. To change the laser wavelength difference in a specific range, one laser wavelength is fixed while the other is variable. The laser wavelength of the first output port of the tunable laser is 1556.776 nm, while laser wavelength of the second varies from 1555.582 nm to 1555.782 nm. In this case, laser wavelength difference varies from 0.994 nm to 1.194 nm, which means that phase difference of the two output signals from the two photodetectors changes from 0.91 π rad to 1.09 π rad on the condition that the *FSR* of the EFPI acoustic sensor is 2.188 nm. Two acoustic signals with different SPLs are applied to the EFPI acoustic sensor. SPLs are set the same as for the experiment on optical power response. Similar results are obtained at these two different conditions. The reference error of the demodulated phase amplitude varies along with the laser wavelength and almost has a linear relationship. The coefficient between the reference error of the demodulated phase amplitude and the reference error of the phase difference from π is calculated to be 1.83, which means when the reference error of the phase difference from π is 1%, the reference error of the demodulated phase amplitude will be 1.8%. The further the phase difference deviates from π, the larger the reference error is. When the phase difference deviates from π, the scatter diagram of those two signals will no longer be a straight line but a curve, which introduces a great error to the linear fitting algorithm. In this case, to lower the reference error of the demodulated phase amplitude, the laser wavelength difference should be strictly controlled to ensure that phase difference is as close as to π as possible.

## 4. Conclusions

In summary, a LF-TIT-DCM-based passive homodyne phase demodulation is proposed. It is able to demodulate phase signals with a large dynamic range and wide frequency band. A detailed theoretical deduction is performed to illustrate each step of the proposed LF-TIT-DCM-based passive homodyne phase demodulation. A dual wavelength phase demodulation system is built to utilize the proposed LF-TIT-DCM algorithm. Experimental results show that it is not only able to demodulate large signals but also small signals. A comparison is made between the LF-TIT-DCM-based passive homodyne phase demodulation and the EF-DCM-based passive homodyne phase demodulation and experimental results show that the linear region of the LF-TIT-DCM-based passive homodyne phase demodulation is much wider than that of the EF-DCM-based passive homodyne phase demodulation, especially in the small signal demodulation. Moreover, the influence of optical power and laser wavelength are researched. Demodulated phase amplitude is hardly influenced by optical power no matter how large the phase amplitude is. On the contrary, since the laser wavelength affected the demodulated phase amplitude indirectly through influencing the phase difference of the two signals from the two photodetectors, the laser wavelength should be strictly controlled to ensure that phase difference is as close as to π as possible.

## Data Availability

The data that support the findings of this study are available from the corresponding author upon reasonable request.
